# Effects of 12-Week Endurance Training at Natural Low Altitude on the Blood Redox Homeostasis of Professional Adolescent Athletes: A Quasi-Experimental Field Trial

**DOI:** 10.1155/2016/4848015

**Published:** 2015-12-10

**Authors:** Tomas K. Tong, Zhaowei Kong, Hua Lin, Yeheng He, Giuseppe Lippi, Qingde Shi, Haifeng Zhang, Jinlei Nie

**Affiliations:** ^1^Department of Physical Education, Dr. Stephen Hui Research Centre for Physical Recreation and Wellness, Hong Kong Baptist University, Hong Kong; ^2^Faculty of Education, University of Macau, Macau; ^3^College of Physical Education, Liaoning Normal University, Dalian, Liaoning 116029, China; ^4^Laboratory of Clinical Chemistry and Hematology, Academic Hospital of Parma, 43126 Parma, Italy; ^5^School of Physical Education and Sports, Macao Polytechnic Institute, Macau; ^6^College of Physical Education, Hebei Normal University, Shijiazhuang, Hebei 050024, China

## Abstract

This field study investigated the influences of exposure to natural low altitude on endurance training-induced alterations of redox homeostasis in professional adolescent runners undergoing 12-week off-season conditioning program at an altitude of 1700 m (Alt), by comparison with that of their counterparts completing the program at sea-level (SL). For age-, gender-, and Tanner-stage-matched comparison, 26 runners (*n* = 13 in each group) were selected and studied. Following the conditioning program, unaltered serum levels of thiobarbituric acid reactive substances (TBARS), total antioxidant capacity (T-AOC), and superoxide dismutase accompanied with an increase in oxidized glutathione (GSSG) and decreases of xanthine oxidase, reduced glutathione (GSH), and GSH/GSSG ratio were observed in both Alt and SL groups. Serum glutathione peroxidase and catalase did not change in SL, whereas these enzymes, respectively, decreased and increased in Alt. Uric acid (UA) decreased in SL and increased in Alt. Moreover, the decreases in GSH and GSH/GSSG ratio in Alt were relatively lower compared to those in SL. Further, significant interindividual correlations were found between changes in catalase and TBARS, as well as between UA and T-AOC. These findings suggest that long-term training at natural low altitude is unlikely to cause retained oxidative stress in professional adolescent runners.

## 1. Introduction

Increasing evidence suggests that exposure to high altitude results in increased production of reactive oxygen species (ROS) from various sources [1]. These essentially include autooxidation of mitochondrial complexes resulting from hypoxia-induced reductive stress [2]; increased conversion of xanthine dehydrogenase to xanthine oxidase [3]; and unprotected exposure to increased ultraviolet radiation [4]. Likewise, downregulation of enzymatic and nonenzymatic antioxidants was found to be associated with high altitude exposure, thus suggesting that the efficiency of the antioxidant system is disrupted simultaneously under the hypoxic environment [5, 6]. Alterations in redox homeostasis could mediate oxidative stress and trigger damage to lipids, proteins, and DNA, which together could ultimately promote cellular degeneration up to apoptosis [7]. Despite these challenges, ROS are crucial for activation of key transcription factors in cells to induce various physiological responses such as mitochondrial biogenesis, angiogenesis, upregulation of antioxidant defense, and oxidative damage-repairing systems, thus favoring health-promoting adaptations in individual [8].

Physical exercise at altitude might lead to exacerbation of altitude-induced oxidative stress and resultant damage, depending on the hypoxic dose and exercise intensity [5, 9, 10]. Nonetheless, there is a general consensus that training at altitude is an effective ergogenic aid to enhance endurance performance at sea-level in athletes, so that this peculiar type of physiological intervention has been used for more than five decades [11]. Classical altitude training requires the training process of athletes living and training at low to moderate natural altitude, the so-called “live high, train high.” Another approach for athletes is the “live high, train low,” which seems effective to mitigate the deleterious effects of altitude exposure caused by decline of training intensity [12]. Previous studies showed that short-term (≤18 days) “live-high, train-low” led to alterations of redox homeostasis in adult athletes, the degree of which is partly determined by the nature of sports (running versus swimming), training intensity, nutritional status, and hypoxic exposure time [13, 14]. Although longer sojourns to altitude potentially lead to greater disturbances in redox balance, relevant researches particularly focused on alterations in redox homeostasis in athletes involved in classical approach with relatively long training period (≥3 months) are limited.

Adolescent athletes participating in training of professional sports (e.g., long-distance running) are increasingly frequent [15]. These athletes are conventionally involved in year-round single-sport training, with a training volume that is globally comparable to that of adult athletes (i.e., 1.5 to 3 training hours per session, two sessions per day, 6 days per week). The training of professional sports specifically which has taken place at natural altitude during certain mesocycle of year-round training is also a common strategy to improve endurance performance in adolescent athletes. Although we have noted well-maintained resting blood redox balance in adolescent athletes participating in professional endurance training and the training regime is not likely to jeopardize the development of their antioxidant defense [16, 17], previous findings have no implications on the alteration of redox homeostasis in adolescent athletes participating in training at natural altitude for a prolonged period. In fact, adolescent athletes are more susceptible than adults to exercise-induced oxidative stress, due to less efficient endogenous antioxidative defense system [18] and more reliance on aerobic metabolism when subjected to identical bulks of physical exercise [19, 20]. Therefore, the aim of this study was to investigate the influence of 12-week exposure to natural altitude on the endurance training-induced alterations in basal redox state of professional adolescent athletes. We hypothesized that the long-term training at natural altitude might generate different adaptations in antioxidant defenses in adolescent athletes compared to their counterparts trained at sea-level, to counteract the potential disturbance in redox balance. At variance with previous investigations, this is the first study to assess the redox balance in adolescent athletes in response to long-term training at natural altitude in an applied field condition. It is known that altitude exposure and exercise are potential sources of oxidative stress [1]. Understanding parameters that regulate the magnitude and duration of the stress response may have a favorable revenue on health and fitness of young athletes. The current findings would hence carry significant implications for those who work with adolescents in this particular profession.

## 2. Methods

### 2.1. Participants

Fifty-three adolescent long-distance runners belonging to a sports club at Liaoning province, China, participated in this study. Selection criteria included the facts that (a) participants were professional trainees in the sports club, (b) they competed at national junior level, (c) they had no family history of cardiovascular disease, nor were they assuming related medication, and (d) they received no anti-inflammatory medications or nutritional supplements. Following an explanation of the purpose and constraints of the study, participants and their guardians gave written informed consent for participating in this trial. The local Ethical Committee for the Use of Human and Animal Participants in Research provided ethical approval of the study.

During the off-season conditioning period, 35 athletes were assigned to have training in the training camp at low altitude (Alt) for 12 weeks, while the rest were trained in a different training camp at sea-level (SL). The separation into two groups was a matter of administration in athletes' affiliated sports club. For gender-, age-, and Tanner-stage-matched comparison, 26 runners trained at either Alt or SL (10 males and 3 females from each) were selected for investigation. The physical characteristics and the information of the sports participation of the selected athletes are shown in Table 1.

### 2.2. Study Design

The alterations of resting serum redox status in response to the 12-week off-season conditioning program in Alt and SL groups were examined. The oxidant and antioxidant status was determined by quantifying serum concentrations of thiobarbituric acid reactive substances (TBARS), total glutathione (T-GSH), reduced glutathione (GSH), oxidized glutathione (GSSG), uric acid (UA), and total antioxidant capacity (T-AOC), as well as the enzymatic activity of xanthine oxidase (XO), glutathione peroxidase (GSH-PX), superoxide dismutase (SOD), and catalase (CAT).

### 2.3. 12-Week Off-Season Conditioning Program

During the study period, Alt runners were living and training at the training camp located at low altitude of 1,700 m in Yunnan, while SL groups were at a different training camp at sea-level in Sichuan, China. Coaches and runners were not informed of the investigation. The conditioning programs adopted in runners in both Alt and SL groups were profession-oriented throughout the investigation period and were not manipulated for investigation purpose.  Table 2 shows the protocol of the 12-week conditioning program used by both groups. Moderate- and high-intensity running training including strength and calisthenics sessions were applied to runners for enhancing endurance performance. The first four weeks was categorized as one microcycle, with training load started relatively easily in 1st week, gradually increasing at 2nd–4th weeks. The increased training load was then maintained in the subsequent 5th–8th weeks and decreased slightly in 9th–12th weeks for preventing overtraining. On each Wednesday, the training load was reduced for recovery purpose. Athletes were left totally free from exercise training on Sunday.

All runners in Alt and SL groups completed the 12-week off-season conditioning program by strictly following the training plan specifically assigned. The training regime was modified slightly to take into account gender differences in physical capability. During the conditioning period, most of the running exercises were held at outdoors. The daytime outdoor temperature at location of both training camps was similar, around 14°C. Due to brutal intensity of sunlight struck on land, the morning training session of Alt group was usually held before sunrise in order to reduce the risk of sun injury. The training in the morning in SL group was generally carried out one hour following breakfast. During the conditioning period, only minor cases of physical injury and sickness were reported in Alt group, and none of these affected the training of the athletes. For the SL group, several athletes ought to be excused from training due to gastrointestinal distress. However, recovery was attained within two days with medication prescribed from team physician. Runners in both groups felt well during the training period but reported being exhausted at the final stage.

The training volume and impulse during the conditioning period of both groups are shown in Table 3. The training volume between the two groups was similar except that the running distance in the first week was comparably lower in Alt group for acclimatization purpose. The training impulse for the conditioning program was evaluated using the session RPE method [21]. The integrated training load in each session was calculated by multiplying duration and intensity (1–10 Borg Rating of Perceived Exertion (RPE) scale) of the session. The average of the arbitrary values per day was slightly higher in Alt group compared to that of SL.

During the camp, the daily food intake of Alt and SL groups was well-controlled and maintained under close surveillance by the same team dietician. The food intake of both groups had never been manipulated for antioxidant supplement purpose. The diets of 7 consecutive days of the 4th week of the program were selected for analysis in both groups using the dietary and nutritional analysis system designed for Chinese athletes and general population (National Research Institute of Sports Medicine, China). The results shown in Table 4 suggest that the daily dietary intakes did not differ between the Alt and SL runners.

### 2.4. Procedures

**Figure 1:**
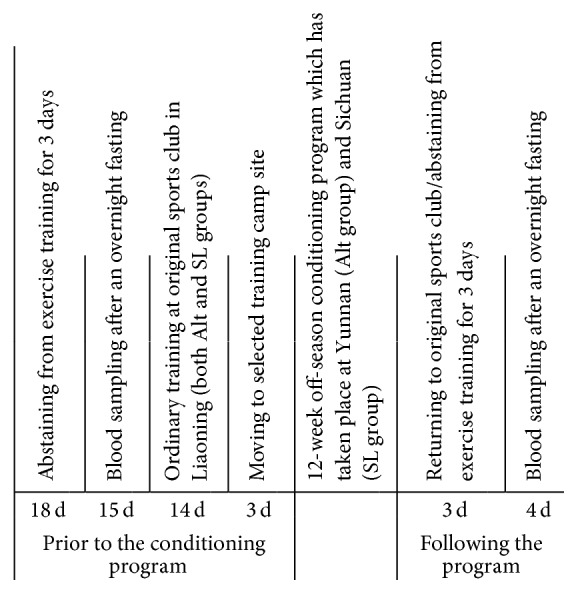
The timeline of the experimental field trial.

For the pre- and postconditioning serum redox profiles, blood sampling was performed two weeks before and three days after the off-season conditioning period, respectively. The timeline of the experimental field trial is shown in Figure 1. The blood samples of Alt and SL groups were collected in the same laboratory at sea-level in Liaoning. Athletes of each group visited the laboratory together, during a single morning session. Prior to each blood sampling, the adolescents were asked to abstain from exercise training for at least 3 days [22]. Upon arrival at the laboratory at 9 am after an overnight fasting, a 10 min rest period was observed. Following anthropometric measurement and Tanner staging assessment, blood samples were collected with athletes in a seated position. A total amount of 5 mL venous blood was drawn from the antecubital vein, using venipuncture for serum redox analyses. The room and board of both groups of runners at Liaoning were uniformed and provided by the sports club. The diets prior to blood sampling were similar before and after conditioning.

### 2.5. Measurements

After blood sampling in vacuum tubes containing no additives, serum was separated at 2,000 g for 20 minutes, aliquoted, and stored at −20°C for later analysis.

The values of TBARS and GSH and the enzymatic activity of XO, SOD, CAT, and UA were measured using commercial assay kits (Nanjing Jiancheng Institute, China) on a spectrophotometer (DU7400, Beckman Co., Fullerton, USA), following manufacturers' instructions. Briefly, lipid peroxidation was evaluated using the thiobarbituric acid reactive substances method and finally expressed as a TBARS concentration [23]. This method was used to obtain a spectrophotometric measurement of the colour produced during the reaction of thiobarbituric acid and malondialdehyde (an indicator of peroxidation of polyunsaturated fatty acids in cell membranes subsequent to reactions with ROS) at 535 nm. The TBARS level was expressed as nmol·mL^−1^.

The values of T-GSH were determined by the glutathione reductase (GR) recycling method [24]. In brief, the GR is reduced to GSH, and then GSH is oxidized to GSSG by DTNB (5,5′-dithiobis(2-nitrobenzoic acid)). The T-GSH was determined by trinitrobenzene, which is the yellow product derived from DTNB, and monitored at 412 nm. In addition, to quantify the amount of GSSG, GSH scavenger was first added into the samples. GSH scavenger eliminates the reduced GSH in the specimen. The samples were also analyzed by the GR recycling method, as for T-GSH above. As one molecule of GSSG could produce two molecules of GSH, the amount of reduced GSH was calculated as T-GSH − (2 × GSSG), and the ratio of reduced and oxidized glutathione was calculated as GSH/GSSG. The T-GSH, GSSG, and GSH are expressed as *μ*mol·L^−1^.

The activity of GSH-PX was measured based on principles described by Hafeman et al. [25]. GSH-PX degraded H_2_O_2_ in the presence of GSH, decreasing GSH levels. The remaining GSH was then measured using the reaction with DTNB. Absorbance was recorded at 412 nm. GSH-PX was expressed in U·mL^−1^, with one unit of GSH-PX enzyme activity being defined as that capable of consuming 1 *μ*mol of GSH per minute.

XO and SOD activities were measured by the xanthine-xanthine oxidase system, which is a superoxide anion generator, following the increase or decrease of absorbance, respectively. The activity of XO and SOD was expressed as U·L^−1^ and U·mL^−1^. CAT activity, expressed as U·mL^−1^, was determined by decrease of H_2_O_2_ absorbance at 240 nm. T-AOC was measured by the ferric reducing ability of plasma (FRAP) assay of Benzie and Strain [26]. The stable colour of the Fe^2+^-o-phenanthroline complex (produced with reducing agents in plasma by reducing Fe^3+^ to Fe^2+^, which reacts with the substrate o-phenanthroline) was measured at 520 nm. T-AOC was expressed in U·mL^−1^, where 1 unit is defined as an increase in absorbance (*A*_520_) of 0.01 per min at 37°C. Finally, UA concentration was determined by an enzymatic method based on the specific uricase-catalyzed oxidation of UA to allantoin and hydrogen peroxide.

The inter- and intra-assay coefficients of variation of the above-mentioned biochemical analyses are as follows: TBARS, 5.4 and 2.2%; XO, 9.2 and 4.5%; SOD, 8.7 and 5.0%; CAT, 11.4 and 6.2%; and T-AOC, 8.5 and 4.6%, UA, 2.8 and 3.0%, T-GSH, 5.7 and 4.8%, and GSH, 4.8 and 1.8%; GSSG, 5.1 and 3.9%, GSH, 4.5 and 4.0%, and GSH-PX, 4.9 and 3.2%, respectively.

### 2.6. Statistical Analysis

Kolmogorov-Smirnov normality test revealed that data for all the variables was normally distributed. Two-way ANOVA with repeated measures was computed to assess differences in serum variables between time points (pre- versus postconditioning) and across groups (Alt versus SL).* Post hoc* analyses using Newman-Keuls were performed for cases in which the main effect was significant. The relationships between variables were assessed using simple linear regression analysis. All tests for statistical significance were standardized at an alpha level of *P* < 0.05, and all results were expressed as mean ± SD.

## 3. Results

Table 5 shows the pre- and postconditioning redox status in serum of Alt and SL groups. Regarding the preconditioning serum redox profile of Alt, the values of serum TBARS and GSH-PX were higher compared to those of SL, while the activities of SOD and GSSG were found to be lower (*P* < 0.05). Following the 12-week conditioning program, serum activities of XO and GSH-PX in Alt were reduced, whereas CAT values increased (*P* < 0.05). Serum TBARS and SOD remained almost unchanged (*P* > 0.05). In SL, serum XO values were also reduced (*P* < 0.05), whereas the interaction of change across the two groups did not achieve statistical significance (*P* > 0.05). Other enzymatic variables and TBARS remained also unchanged in SL during postconditioning (*P* > 0.05). For nonenzymatic variables, serum T-AOC did not change in both groups in the postconditioning period (*P* > 0.05). A modest decrease of serum T-GSH was found in SL during postconditioning, whereas an opposite but nonstatistically significant variation was observed in Alt. The interaction of change in T-GSH across the two groups was significant (*P* < 0.05). In both groups, serum GSSG and GSH were, respectively, increased and reduced during postconditioning (*P* < 0.05). Nevertheless, greater reductions in GSH as well as in the corresponding GSH/GSSG ratio were found in SL compared to Alt (*P* < 0.05). For serum UA, a significant increase was recorded in Alt in the postconditioning period, whereas its concentration was significantly reduced in SL (*P* < 0.05). The interaction of the change in UA across the two groups was significant (*P* < 0.05).

For the changes in the variables expressed as percentage of preconditioning values, significant interindividual correlations were found between serum TBARS and CAT (*r* = 0.41, *n* = 26, *P* < 0.05), as well as between serum T-AOC and UA (*r* = 0.42; *P* < 0.05). Significant correlations were also appreciated between changes of T-GSH and GSH (*r* = 0.58: *P* < 0.05), T-GSH and GSSG (*r* = 0.40; *P* < 0.05), and GSH and GSSG (*r* = −0.39; *P* < 0.05).

## 4. Discussion

This study investigated for the first time the influence of exposure to natural altitude on endurance training-induced alterations of redox homeostasis in professional adolescent runners by comparing the observed changes with those of their counterparts who trained at sea-level. The investigation of redox responses of Alt and SL runners to endurance running workouts was carried out during the 12-week off-season conditioning period of their whole-year training program. The present study is quasi-experimental, as athletes assigned to the Alt and SL groups were not on the random basis despite the fact that the two groups were homogeneous, and their training was under the surveillance of the same sports club.

TBARS, resulting from lipid peroxidation of polyunsaturated fatty acids, is a well-established biomarker of oxidative stress [23]. In this study, the 12-week conditioning program either at SL or at Alt did not alter resting serum TBARS values of runners, thus suggesting that redox homeostasis was well maintained. The current findings of absence of adverse effects on resting blood redox balance in adolescent athletes participating in professional endurance training are in agreement with previous reports of athletes in response to training at sea-level [16, 17]. This evidence is also consistent with a favorable blood redox profile in adolescent athletes in response to long-term endurance training at natural low altitude. Acute exposure to high altitude causing increased lipid peroxidation has been previously reported in animal and human models [1]. Nevertheless, the level of altitude-induced oxidative stress is parallel to the increase in altitude in humans [5]. Despite a lack of data about acute response to altitude, there is general consensus that the oxidative stress acutely elicited in Alt runners due to training at low altitude exposure (1700 m) is mild and not likely to be comparable to that caused by training at high altitude (i.e., 79% increase in lipid peroxidation at 8848 m) [5]. Acclimatization to long-term exposure to altitude could also alleviate the oxidative stress and the associated increase of circulating products of lipid peroxidation [8, 27]. Moreover, regular exercise training could attenuate lipid peroxidation due to training-induced increase in antioxidant capacity and lipid repair [8]. The unaltered TBARS level in Alt runners may be partly attributed to enhancement of individual antioxidant level, as confirmed by the increase in serum CAT and by its significant association (*r* > 0.4) with changes in serum TBARS, thus integrating result of long-term acclimatization to altitude and adaptations to physical training.

In the SL group, the decreases in resting serum XO and UA levels following the 12-week conditioning program are in line with the previous notion that exercise training could modify enzyme activities for decreasing purine metabolism [28, 29]. On the other hand, ischemia in body tissues or exposure to low oxygen pressure would lead to adenine nucleotide degradation by increased XO activity and purine metabolism [30]. The increased conversion of xanthine dehydrogenase (XDH) to XO, and the consequent production of UA and superoxide anion, may be regarded as a potent source of ROS generation during altitude exposure. However, we failed to observe increased values of serum XO in Alt runners after the 12-week conditioning program. This suggests that the XDH/XO system of Alt runners might not have substantially worsened an oxidative load during the long-term low altitude exposure. It has been reported that the role played by conversion of XDH to XO to increased lipid peroxidation was more substantial in intermittent rather than in continuous exposure to altitude, since the former type of acclimatization has characteristics very similar to the ischemia/reperfusion process [31]. The decrease in serum XO observed in Alt runners, with a magnitude similar to that of SL group, possibly results from adaptation to endurance training. Nevertheless, a corresponding decrease in serum UA, which appeared in SL group, was not observed in Alt runners. The increased accumulation of UA in blood may be partly attributed to downregulation of renal excretion, which seems commonplace in individuals with chronic exposure to high altitude [32]. UA, the final product of purine metabolism in humans, is considered a potent scavenger of ROS and represents almost 50% of the T-AOC [7, 33]. The modest increase of serum UA concentration that we have observed in Alt runners can hence be interpreted as a beneficial mechanism aimed to enhance their antioxidant pool. This hypothesis is supported by the significant correlation observed between changes of UA and T-AOC (*r* > 0.4) and would ultimately promote a mechanism of protection against oxidative stress.

The endurance training-induced alterations in serum levels of selected antioxidants were not significant in SL runners after the 12-week conditioning. This is in agreement with the previous notion that adaptive responses of the antioxidant system to endurance training are usually pronounced in subjects with low training level at beginning of the protocol [7, 17]. Information on untrained counterparts is unavailable in the present study, but the levels of antioxidants are generally higher in runners than in age-matched untrained adolescents [16]. Although an oxidative stress mirrored by overaccumulation of TBARS could not be found in SL runners, we observed a modest decrease of T-GSH, accompanied by a marked increase of GSSG, a decrease in GSH, and a resulting reduction of the GSH/GSSG ratio despite the fact that the adolescent runners were forced to abstain from exercise training for three days. As runners' nutrient intake was maintained constant throughout the study period, the persistent suppression of serum GSH/GSSG ratio was possibly due to the counteraction of oxidative stress elicited from physical demands of training, which have been reported in adolescents participating regularly in sports training [22, 34].

Interestingly, the change of GSH system in Alt runners mirrored that of the SL group, although the overall variation was much lower. The mild changes of GSH, GSSG, and GSH/GSSG ratio accompanied by the marked decrease of GSH-PX are seemingly inexplicable since training volume and intensity were not different between Alt and SL groups, whereas Alt runners were expected to develop a protective mechanism against higher level of oxidative stress. Moreover, GSH-PX activity is strongly dependent upon the GSH synthesis of glutamyl cycle [1]. The relative lower decrease of the GSH/GSSG ratio coupled with a significant downregulation of GSH-PX suggests that there might be additional factors other than the state of thiol system that may interfere with enzyme activity. GSH-PX contains selenium as cofactor for reduction [35]. Nutritional deficiency of selenium in the animal-human food chain at elevated global regions has been reported to be a cause of low levels of serum selenium and GSH-PX in local residents [36]. Although the daily alimentary intake of both Alt and SL runners was matched, the foods in the two training camps were sourced from different locations (i.e., altitude versus sea-level). As such, we cannot rule out that the low GSH-PX activity observed in Alt runners may be attributed to selenium deficiency in local foods. GSH-PX, CAT, and SOD are intercellular defense enzymes responsible for counteracting the effects of ROS. SOD is the first line of defense to convert the O_2_^−^ to H_2_O_2_, while GSH-PX and CAT catalyze the conversion of H_2_O_2_ into H_2_O [7]. The peroxidase reaction of GSH-PX utilizes GSH as substrate and appears more efficient in the presence of high ROS concentration [37]. It is known that antioxidant defenses in humans interplay as a coordinated system, with various metabolites and enzymes providing synergistic and interdependent effects. In this study, the level of SOD in serum did not change after the conditioning period, whereas a marked increase was found in serum CAT values. This latter evidence might be interpreted as an interactive outcome of the antioxidant defense in response to the downregulation of GSH-PX, aimed to prevent severe oxidative injury in Alt runners. In line with this hypothesis, patients with stomach cancer at early stages were found to exhibit a similar compensatory defense reaction, involving an increase of CAT activity likely attributable to a decrease of GSH-PX and a resultant increase of GSH [38].

Although our findings provide reasonable information regarding the antioxidant defense against chronic exercise-induced oxidative stress of professional adolescent runners at natural low altitude, further interpretation is constrained by some limitations. First, the changes in serum TBARS do not always reflect the real level of oxidative stress in runners. The TBARS assay is conventionally regarded as a reliable method for measuring lipid peroxidation, but it is not characterized by absolute specificity for this pathway [7]. In fact, changes in the concentration of TBARS were found to be similar to exercise-induced changes in F2-isoprostane concentrations—product of ROS-catalyzed peroxidation of essential fatty acids* in vivo* [39]. Moreover, oxidative stresses assessed with the concentrations of peroxidation products of lipids and proteins were correlated with each other [40]. It is also noteworthy that changes in the limited blood redox markers may not fully reflect the true alterations of the redox status in active tissues. However, tissue biopsy sampling techniques in humans can be only performed in special cases. Nonetheless, the selected serum redox biomarkers including TBARS, CAT, and GSH were proven to be reliable indices that mirror the exercise-induced changes appearing in skeletal muscle, heart, and liver [41]. In this study, only resting blood redox data collected before and after the 12-week conditioning period were available for the analysis, whereas data both at rest and after exercise during the conditioning period were lacking due to the reasonable antagonism of athletes/coaches towards repeated blood sampling in adolescent athletes. The food intake and meal pattern of SL and Alt groups were matched, but daily nutrient intake of athletes had not been thoughtfully monitored. However, we assume that antioxidant dietary intakes were similar between the two groups. In future studies, the assessment of plasma antioxidant vitamins and Trolox-equivalent antioxidant capacity may provide additional information about antioxidant defense capacity in athletes. Finally, the changes observed in the athletes of both SL and Alt groups were independent of their sex hormones. Although the changes in estrogen level during the menstrual cycle are considered of minor influence on exercise-induced oxidative stress in young women [42], testosterone has been shown to inhibit neutrophil prooxidative capability and increase the content of thiol groups* in vitro* [43]. Puberty tends to upregulate the antioxidant capacity of adolescents [44]. It is advisable to consider the potential effect of the sex hormones in adolescent athletes in future studies.

In conclusion, the 12-week off-season conditioning program at sea-level or natural low altitude of 1700 m does not seem to cause a permanent oxidative stress in professional adolescent endurance runners. The exaggerated challenge in maintenance of redox homeostasis in runners trained at altitude was probably counterbalanced by an integrated effect of individual antioxidants enhancement, which can hence be considered an adaptive mechanism to professional endurance training and long-term acclimatization to altitude. The present findings complement and extend previous observations that natural low altitude training of professional adolescent runners, with training volume comparable to that of adult athletes, is not likely to jeopardize the development of their antioxidant defense system for protection against oxidative damage [16, 17].

## Figures and Tables

**Table 1:** Physical characteristics and sports participation of altitude (Alt) and sea-level (SL) runners.

	Alt [*n* = 13 (10 males, 3 females)]	SL [*n* = 13 (10 males, 3 females)]
Age (yrs)	15.0 ± 1.3	14.7 ± 1.9
Tanner stage	4.5 ± 0.7	4.2 ± 0.8
Weight (kg)	58.2 ± 3.6	58.0 ± 1.6
Height (cm)	173.2 ± 6.2	172.4 ± 4.4
BMI (kg/m^2^)	19.4 ± 0.8	19.5 ± 0.9
VO_2max_ (mL·kg^−1^·min^−1^)	61.2 ± 4.8	60.1 ± 6.5
Years of training	3.2 ± 0.5	2.9 ± 0.7
Number of races per year	4–7	4–7

Values are mean ± SD.

**Table 2:** The protocol of the 12-week off-season conditioning program.

Day	Monday	Tuesday	Wednesday	Thursday	Friday	Saturday	Sunday
Running pace	Moderate	Fast-race	Slow	Fast	Fast-race	Moderate	
Week							
1	Short—9.5 km	Long—19.5 km	Short—13 km Calisthenics	Short—13 km Strength	Short—9.5 km Interval 4 × 800 m	Short—13 km Strength	Rest
2	Short—13 km	Long—22.5 km	Short—9.5 km Strength	Long—16 km Calisthenics	Short—9.5 km Interval 4 × 1000 m	Long—16 km Strength	Rest
3	Long—16 km	Long—25.5 km	Short—13 km Strength	Short—13 km Calisthenics	Short—13 km Interval 10 × 400 m	Short—9.5 km Strength	Rest
4	Short—9.5 km	Long—16 km	Short—13 km Strength	Short—13 km Calisthenics	Short—13 km Interval 6 × 400 m	Short—13 km Strength	Rest
5	Long—16 km	Long—22.5 km	Short—13 km Strength	Long—16 km Calisthenics	Short—13 km Interval 4 × 800 m	Long—16 km Strength	Rest
6	Short—13 km	Long—25.5 km	Short—13 km Calisthenics	Long—16 km Calisthenics	Short—13 km	Short—16 km Strength	Rest
7	Short—13 km	Long—29 km	Short—13 km Calisthenics	Strength	Short—13 km Interval 6 × 400 m	Long—25.5 km	Rest
8	Short—13 km	Long—29 km	Short—13 km Calisthenics	Short—9.5 km Interval 6 × 400 m	Short—13 km	Long—16 km Strength	Rest
9	Short—13 km	Long—25.5 km	Short—13 km Calisthenics	Rest	Short—13 km Strength	Long—16 km	Rest
10	Short—13 km	Long—29 km	Short—9.5 km Strength	Long—16 km Calisthenics	Short—13 km Interval 8 × 200 m	Long—19.5 km	Rest
11	Short—13 km	Long—25.5 km	Short—13 km Calisthenics	Short—13 km	Short—13 km Interval 6 × 200 m	Long—16 km Strength	Rest
12	Short—13 km	Long—25.5 km	Short—13 km Strength	Rest	Short—13 km Interval 4 × 200 m	Long—19.5 km Strength	Rest

**Table 3:** The training volume and impulse of altitude (Alt) and sea-level (SL) runners during the 12-week off-season conditioning program.

	Alt (*n* = 13)	SL (*n* = 13)
Training volume		
Number of session/day	2	2
Training hours/session	1.5	1.5
Training days/week	6	6
Training impulse/day	967.0 ± 14.4	845.8 ± 11.6^a^

Values are mean ± SD.

^a^Significantly different from corresponding Alt value, *P* < 0.05.

**Table 4:** Daily dietary intakes of altitude (Alt) and sea-level (SL) runners during the 12-week off-season conditioning program.

	Alt (*n* = 13)	SL (*n* = 13)
Total energy intake (Kcal)	2311.7 ± 241.5	2397.2 ± 241.7
Protein (g)	81.0 ± 6.3	85.2 ± 11.2
Protein (%EI)	14.1 ± 0.9	14.2 ± 1.5
CHO (g)	364.6 ± 48.0	367.4 ± 55.9
CHO (%EI)	63.0 ± 3.7	61.1 ± 4.3
Fat (g)	58.9 ± 12.2	65.3 ± 8.3
Saturated fat (g)	8.7 ± 1.4	10.0 ± 2.6
Monounsaturated fat (g)	15.8 ± 1.8	20.7 ± 3.0
Polyunsaturated fat (g)	10.7 ± 1.0	12.0 ± 3.2
Cholesterol (mg)	262.3 ± 41.2	243.8 ± 64.8
Fat (%EI)	23.0 ± 3.9	24.7 ± 3.7
Fibres (g)	12.1 ± 2.8	11.2 ± 2.0
Vitamin A (*μ*g RE)	695.0 ± 270.2	730.6 ± 246.7
Vitamin C (mg)	91.5 ± 49.2	81.0 ± 32.5
*α*-Tocopherol (mg)	27.7 ± 6.8	29.9 ± 7.4
Selenium (*μ*g)	76.4 ± 9.8	87.3 ± 14.4
Zinc (mg)	13.4 ± 2.2	13.6 ± 1.9
Copper (mg)	2.42 ± 0.35	2.32 ± 0.44
Iron (mg)	28.2 ± 5.0	27.0 ± 4.8
Magnesium (mg)	346.5 ± 37.9	332.5 ± 42.8
Manganese (mg)	7.60 ± 1.41	7.10 ± 1.05

Values are mean ± SD.

EI: total energy intake; CHO: carbohydrate.

**Table 5:** Resting levels of serum thiobarbituric acid reactive substances (TBARSs), xanthine oxidase (XO), total antioxidant capacity (T-AOC), superoxide dismutase (SOD), catalase (CAT), glutathione peroxidase (GSH-PX), total glutathione (T-GSH), oxidized glutathione (GSSG), reduced glutathione (GSH), GSH/GSSG ratio, and uric acid (UA) of altitude (Alt) and sea-level (SL) runners before (pre) and after (post) the 12-week off-season conditioning program.

	Alt (*n* = 13)		SL (*n* = 13)	
	Before	After	Before	After
TBARS (nmol·mL^−1^)	4.81 ± 1.15	4.46 ± 1.22	2.62 ± 1.02^a^	2.17 ± 0.80^a^
XOD (U·L^−1^)	20.8 ± 4.2	15.9 ± 4.9^*∗*^	18.2 ± 6.5	12.4 ± 3.4^*∗*^
T-AOC (U·mL^−1^)	14.6 ± 3.2	14.5 ± 7.2	14.1 ± 2.8	12.4 ± 3.8
SOD (U·mL^−1^)	55.6 ± 18.6	55.6 ± 13.6	77.7 ± 23.0^a^	67.8 ± 21.6
CAT (U·mL^−1^)	1.48 ± 0.50	2.16 ± 0.60^*∗*^	1.46 ± 0.60	1.63 ± 0.67^a†^
GSH-PX (U·mL^−1^)	114.5 ± 24.1	88.1 ± 32.8^*∗*^	89.8 ± 21.4^a^	73.1 ± 38.2
T-GSH (*μ*mol·L^−1^)	17.5 ± 1.1	18.5 ± 2.4	18.2 ± 1.3	17.1 ± 1.2^*∗*†^
GSSG (*μ*mol·L^−1^)	5.31 ± 0.75	6.41 ± 0.93^*∗*^	5.96 ± 0.46^a^	7.01 ± 0.87^*∗*^
GSH (*μ*mol·L^−1^)	6.88 ± 1.60	5.65 ± 1.29^*∗*^	6.31 ± 1.31	3.11 ± 1.50^*∗*a†^
GSH/GSSG	1.37 ± 0.62	0.89 ± 0.21^*∗*^	1.07 ± 0.26	0.47 ± 0.28^*∗*a†^
UA (mg·L^−1^)	34.3 ± 9.0	38.1 ± 7.5^*∗*^	31.3 ± 5.5	28.1 ± 5.2^*∗*a†^

Values are mean ± SD.

^*∗*^Significantly different from corresponding preconditioning value, *P* < 0.05.

^a^Significantly different from corresponding Alt value, *P* < 0.05.

^†^Conditioning-induced change is significantly different from corresponding Alt value, *P* < 0.05.
